# Mechanism for the Potential Inhibition Effect of Microcystin-LR Disinfectant By-Products on Protein Phosphatase 2A

**DOI:** 10.3390/toxins14120878

**Published:** 2022-12-16

**Authors:** Huiqun Yu, Yixue Xu, Jiyuan Cui, Wansong Zong

**Affiliations:** College of Geography and Environment, Shandong Normal University, 88# East Wenhua Road, Jinan 250014, China

**Keywords:** microcystin-LR, disinfection by-products, protein phosphatase 2A, molecular docking, molecular mechanism

## Abstract

The secondary contamination of microcystin disinfection by-products (MC-DBPs) is of concern due to the residual structure similar to their original toxin. Based on identification and preparation, the potential inhibition effect of typical MCLR-DBPs (associated with the oxidation of Adda^5^) on PP2A was confirmed in the sequence of MCLR > P1 > P4 > P3 ≈ P2 > P7 ≈ P6 ≈ P5 > P8. To elucidate the molecular mechanism underlying the inhibition effect, the interaction models for typical MCLR-DBPs and PP2A were constructed using a modeling-based-on-ligand-similarity approach, and the candidate interaction parameters between typical MCLR-DBPs and PP2A were obtained by molecular docking. By analyzing the correlation between inhibition data and candidate interaction parameters, the key interaction parameters were filtered as hydrogen bonds “Adda^5^”←Asn_117_, “Adda^5^”←His_118_, MeAsp^3^←Arg_89_, Arg^4^←Arg_214_, Arg^4^→Pro_213_; ionic bonds Glu^6^-Arg_89_, Asp_85_-Mn_1_^2+^, Asp_57_-Mn_2_^2+^; and metal bonds Glu^6^-Mn_1_^2+^, Glu^6^-Mn_2_^2+^. With the gradual intensification of chlorination, Adda^5^ was destroyed to varying degrees. The key interactions changed correspondingly, resulting in the discrepant inhibition effects of typical MCLR-DBPs on PP2A.

## 1. Introduction

Microcystin (MC) pollution related to eutrophication has become a widespread concern of researchers around the world [1]. As the secondary metabolites from algal blooms, MCs are released into the water after algae cells die and rupture. MCs not only affect water quality, but also pose a serious threat to water ecosystems and public health and safety [2]. MCs are a class of cyclic heptapeptides that share the common structure cyclo-D-Ala^1^-X^2^-D-isoAsp^3^-Z^4^-Adda^5^-D-isoGlu^6^-Mdha^7^ [3]. Due to variable amino acids at position X^2^/Z^4^ and methylation of other residues, more than 100 different MC isomers have been identified [4]. Among these MCs, MCLR is the most toxic and widespread isomer [5].

MCs have strong hepatotoxicity [6,7]. Acute poisoning is mainly manifested in liver redness, swelling and bleeding, destruction of the hepatocyte skeleton, and so on [8]. The toxic mechanism of MCs involves the inhibition of protein phosphatases (PPs, the main regulatory factors of protein dephosphorylation) [9]. Crystal structure analysis for MCLR-PPs complexes revealed that MCs undergo a two-step interaction with PPs [10,11]: in the first step, partial surface hydrophobic amino acid residues form a cage structure by hydrophobic interaction and quickly envelop the side chain of Adda^5^; in the second step, the C=C bond of Mdha^7^ is irreversibly bound to specific nucleophilic sites (typically cysteine residues) by electrophilic addition reaction. Finally, the important interactions between the conserved domains of PPs (especially nine strictly conserved amino acids) and metal ions interacting with the introduced phosphate group change accordingly, resulting in the inhibition of PPs activity [12]. Consequently, the intracellular phosphorylated functional protein is overexpressed [13,14], resulting in massive hemorrhage, swelling, and necrosis of mammalian liver cells [15].

To control the environmental risks of MCs, disinfection technology has been widely adopted to control MC-contaminated water and this exhibited obvious technical advantages [16,17]. MCs can be decomposed into low-toxicity or non-toxic substances by disinfectants that destroy the critical structures [18,19]. However, these processes might produce a variety of primary MC-related disinfection by-products (MC-DBPs) [19], which might retain the original toxic groups and thus produce potential inhibition effects on PPs [20,21].

A ligand–receptor interaction model could help extract the interaction parameters and explore the key interaction process. At present, information on the interactions between MC-DBPs and PPs is limited and interaction models for MC-DBP-PP complexes are not available. The relationship between the residual structure of MC-DBPs and biotoxicity is therefore not clear, and the mechanism for the potential inhibition effect of MC-DBPs on PPs is difficult to elucidate. The pioneering study of Xu et al. [22] provided a new viewpoint on evaluating the interactions between structural analogues and proteins without corresponding interaction models. They constructed interaction models for typical MC and PP2A complexes based on modeling ligand similarity and explored the important interactions based on molecular docking. The ligand-similarity approach not only helped to fill the gaps in the interaction models of MC structural analogs and proteins, but also explored the important interaction parameters, which was conducive to the study of the molecular mechanism.

In view of this, the modeling-based-on-ligand-similarity strategy and molecular docking simulation were adopted and integrated herein to explore the molecular mechanism of the potential inhibition effect of typical MCLR-DBPs on PP2As (the most important PPs in eukaryotic cells). Based on typical MCLR-DBPs derived from chlorine disinfection in the identification and preparation process, their inhibition effects on PP2A were evaluated by a traditional PPs inhibition assay [19]. With the assistance of molecular simulation, the interaction models for typical MCLR-DBPs and PP2A were constructed by modeling ligand similarity based on the crystal structure of the MCLR-PP2A complex. Subsequently, the candidate interaction parameters such as related areas, hydrogen bonds, metal bonds, and ionic bonds between the toxins and PP2A were obtained based on molecular docking simulation. By analyzing the correlation between inhibition data and candidate interaction parameters, the key interaction parameters were filtered. Further 2D ligand interaction analysis revealed the key sites and the key interactions between typical MCLR-DBPs and PP2A. Taking the key interactions into consideration, the molecular mechanism for the potential inhibition effect of typical MCLR-DBPs on PP2A was clarified in detail. Compared with the study of Xu et al. [22], our study considered that the disinfection process might affect the interactions between nine conserved amino acids of PP2A and Mn^2+^ions/the introduced phosphate group, thus further explored the molecular mechanism for the potential inhibition effect of typical MCLR-DBPs on PP2A. Additionally, the analysis of the molecular mechanism was more comprehensive than that of Xu et al. [22]. We considered that the changed interactions affected by the disinfection process might be divided into multiple stages. Finally, the conditions and applications of the template docking mode were emphasized in the study herein. The current study draws increased attention to the secondary pollution caused by MC-DBPs. This study also contributes to development of an MCs regulation strategy, and therefore has important theoretical and practical application value.

## 2. Results and Discussions

### 2.1. Typical MCLR-DBPs Identification Based on MS and HPLC Analysis

**Figure 1:**
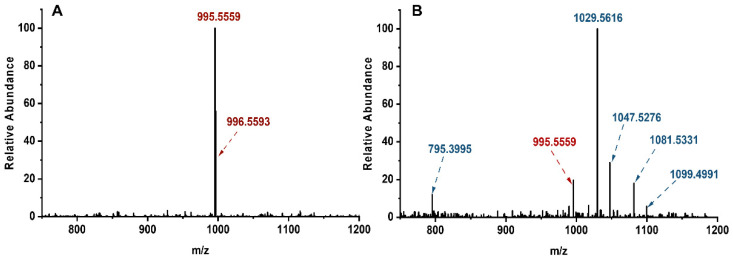
MS spectra for MCLR (**A**) and its disinfection sample (**B**) subject to chlorination for 40 min.

Subject to chlorination, MCLR might convert into multiple typical MCLR-DBPs with different molecular weights that could be detected with a mass spectrometer (Figure 1). For MCLR (C_49_H_74_N_10_O_12_), its major MS signals could be detected at *m*/*z* 995.5559 and 996.5593 (the chief isotopic peak), corresponding to the singles of protonated toxin (Figure 1A). For the disinfection sample, five newly formed MS signals for single protonated typical MCLR-DBPs could be detected at *m*/*z* 1047.5276, 1029.5616, 1099.4991, 1081.5331, and 795.3995 (Figure 1B). With the assistance of Compass Isotope Pattern software, the chemical formulas for the above MCLR-DBPs could be identified as C_49_H_75_N_10_O_13_Cl (+OH, +Cl), C_49_H_76_N_10_O_14_ (+2OH), C_49_H_76_N_10_O_14_Cl_2_ (+2OH, +2Cl), C_49_H_77_N_10_O_15_Cl (+3OH, +Cl), and C_34_H_54_N_10_O_12_ (−15C, −20H).

**Figure 2:**
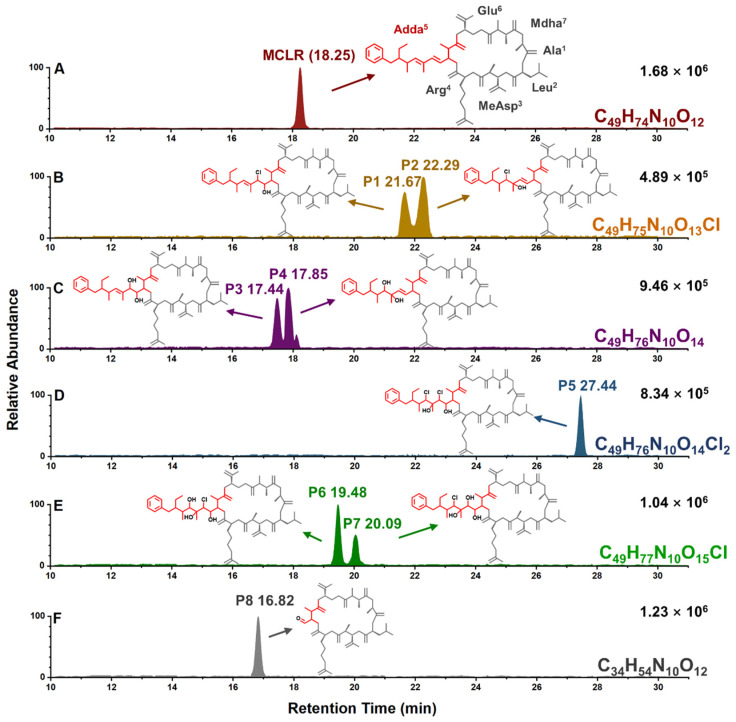
Extract ion chromatograms of native MCLR and typical MCLR-DBPs from disinfection sample (40 min, with their separate max. abundances fixed at 100%). Extract ion chromatograms of native MCLR (**A**), P1 and P2 (**B**), P3 and P4 (**C**), P5 (**D**), P6 and P7 (**E**), P8 (**F**).

After solid-phase extraction, the preliminarily detected typical MCLR-DBPs in crude extract were purified by preparative chromatography separation. Simultaneously, the possible isomers for above MCLR-DBPs (with identical MS signals) could be separated and identified according to their extract ion chromatogram (EIC) peaks (Figure 2) and characteristic MS/MS fragments. For MCLR, it had a EIC peak around 18.25 min (Figure 2A) and several corresponding MS/MS fragments at *m*/*z* 213.0834, 286.1479, 553.3072, 599.3546, 682.3959, and 866.5150. With the assistance of Compass Isotope Pattern software, the typical MS/MS fragments could be identified as [Glu^6^-Mdha^7^ + H]^+^, [MeAsp^3^-Arg^4^ + H]^+^, [Mdha^7^-Ala^1^-Leu^2^-MeAsp^3^-Arg^4^ + H]^+^, [MeAsp^3^-Arg^4^-Adda^5^ + H]^+^/[Arg^4^-Adda^5^-Glu^6^ + H]^+^, [Arg^4^-Adda^5^-Glu^6^-Mdha^7^ + H]^+^, and [Mdha^7^-Ala^1^-Leu^2^-MeAsp^3^-Arg^4^-Adda^5^ + H]^+^/[Arg^4^-Adda^5^-Glu^6^-Mdha^7^-Ala^1^-Leu^2^ + H]^+^ [20].

For typical MCLR-DBPs, eight EIC peaks were eluted between 16.82 min and 27.44 min (Figure 2A–F). By comparing the MS/MS fragments related to the newly formed EIC peaks with that of MCLR, it could be determined that Adda^5^ was the main reaction site (Table S1). Combined with chemical formula analysis, the generative mechanisms for the typical MCLR-DBPs were proposed: MCLR-DBP C_49_H_75_N_10_O_13_Cl had two isomers that were eluted at 21.67 min (P1) and 22.29 min (P2) (Figure 2B). P1/P2 should be the addition products of 1·Cl + 1·OH to the conjugated diene of Adda^5^. According to Markovnikov’s rule, Cl should be added to the C atom with more H atoms, while ·OH should be added to the C atom with less H atoms. On account of steric hindrance theory, the addition product to the inner double bond should have a lower abundance than the addition product to the external double bond. From this, P1 and P2 should be the addition product to the inner double bond and the addition product to the external double bond, respectively. For MCLR-DBP C_49_H_76_N_10_O_14_ with two EIC peaks at 17.44 min and 17.85 min (Figure 2C), it could be formed by the addition of 2·OH to the conjugated diene of Adda^5^ or transformed by substituting 1·Cl in the conjugated diene of P1/P2 with ·OH. Similarly, the addition product of the inner double bond (P3) should have a lower abundance than the external double bond (P4) on account of steric hindrance theory. For MCLR-DBP C_49_H_76_N_10_O_14_Cl_2_ with only one EIC peak at 27.44 min (Figure 2D), 2·Cl+2·OH should be successively added to the two double bonds and thus formed P5. Likewise, P5 also could be transformed from P1/P2 by adding 1·Cl + 1·OH to another C=C bond in Adda^5^. For MCLR-DBP C_49_H_77_N_10_O_15_Cl with two EIC peaks at 19.48 min and 20.09 min (Figure 2E), there should be two MCLR-DBP isomers P6/P7, which should be the secondary products of P1/P2/P3/P4. Taking the abundances of P1/P2/P3/P4 into account, P6 (the isomer with higher abundance) should be the secondary product of P1 and P4, while P7 (the isomer with lower abundance) should be the secondary product of P2 and P3. For MCLR-DBP C_34_H_54_N_10_O_12_ with only one EIC peak at 16.82 min (Figure 2F), the decreased molecular weight meant 15C+20H were removed from the side chain of Adda^5^ and 1C+1H+1O were left (P8). P8 could also be transformed from MCLR/P1/P3/P5/P6/P7 by the oxidation of inner double bonds, forming C=O bonds.

### 2.2. Potential Inhibition Effect for Typical MCLR-DBPs Target to PP2A

**Figure 3:**
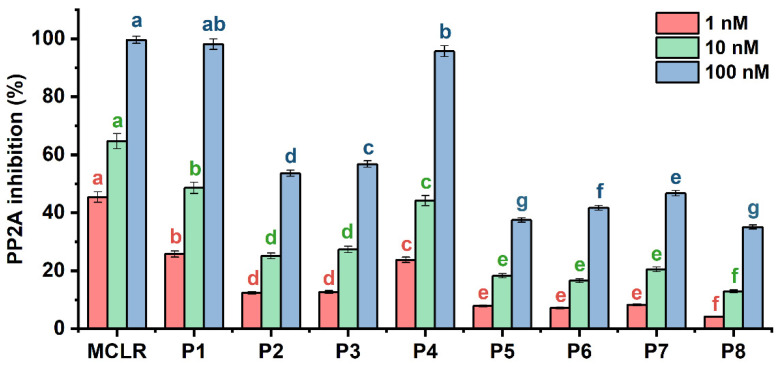
The inhibition effect of MCLR and typical MCLR-DBPs on PP2A. The error bar is the standard error of three repeated analyses. A one-way analysis of variance (ANOVA) followed by LSD post-hoc tests were used to verify significant differences among MCLR and MCLR-DBPs. Different letters indicate significant differences between groups (*p* < 0.05) obtained using SPSS software [23].

Eluted typical MCLR-DBPs were collected around their specific retention times. The preparation and purification information for typical MCLR-DBPs was listed in Table S2. Due to their higher purity (>98.6%), the prepared MCLR-DBP samples were directly used in the PP2A inhibition assay. According to Figure 3, all of the typical MCLR-DBPs exhibited inhibition effects on PP2A. Compared with MCLR, the inhibition effect of typical MCLR-DBPs decreased in different degrees. At 1 nM and 10 nM, the inhibition effect of toxins could be divided into six categories (a MCLR; b P1; c P4; d P2, P3; e P5, P6, P7; and f P8) according to ANOVA. At 100 nM, the inhibition effect of toxins could be divided into seven categories according to ANOVA: (a) MCLR; (ab) P1; (b) P4; (c) P3; (d) P2; (e) P7; (f) P6; and (g) P5, P8. To sum up, the inhibition sequence could be identified as MCLR > P1 > P4 > P3 ≈ P2 > P7 ≈ P6 ≈ P5 > P8. Basically, the inhibition effect of MCLR-DBPs on PP2A gradually decreased as the reaction progressed. What should be emphasized is the considerable inhibition effect of MCLR-DBPs (especially P1, P4) on PP2A; their secondary environmental risk could not be ignored.

### 2.3. Simulation for the Interactions between Typical MCLR-DBPs and PP2A Based on Ligand Similarity Modeling and Molecular Docking

**Figure 4:**
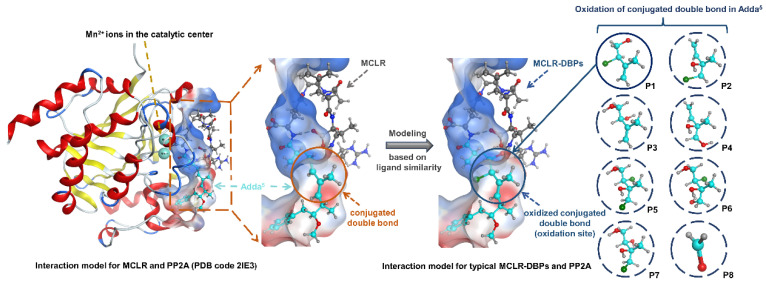
Illustration for interaction model construction for typical MCLR-DBP-PP2A complexes (with no PDB models) based on the ligand-similarity-modeling strategy.

The potential inhibition effect of typical MCLR-DBPs on PP2A should be attributed to their residual toxic groups derived from MCLR. As limited information was available for the interactions between MCLR-DBPs and PP2A, it was difficult to evaluate the potential inhibition effect of typical MCLR-DBPs on PP2A. Since MCLR and MCLR-DBPs were considered to have a reasonable structural similarity, MCLR was an ideal template to construct MCLR-DBPs. With the assistance of molecular simulation, the models for typical MCLR-DBPs and PP2A were constructed by modeling based on ligand similarity (see Figure 4). The model for MCLR-PP2A was obtained from the Protein Data Bank (PDB code 2IE3) and preprocessed by “building missing loops” and adjusting the charges of the whole system. Based on the revised model for MCLR-PP2A, the models for typical MCLR-DBPs and PP2A complexes could be preliminarily constructed through “modeling based on ligand similarity”: the original ligand MCLR in the revised model was replaced by the identified MCLR-DBPs. Then, the models for MCLR-DBPs and PP2A complexes were energy minimized and re-docked though “template dock” mode to ensure the reliability of the models. On this basis, 80 candidate interaction parameters (combination areas, related surface areas, related chemical bonds) between toxins and PP2A, the exposure areas of the catalytic center, and related parameters for toxins were obtained with molecular docking simulation (listed in Table S3).

### 2.4. Pearson Correlation Analysis for the Candidate Interaction Parameters and Inhibition Data

**Figure 5:**
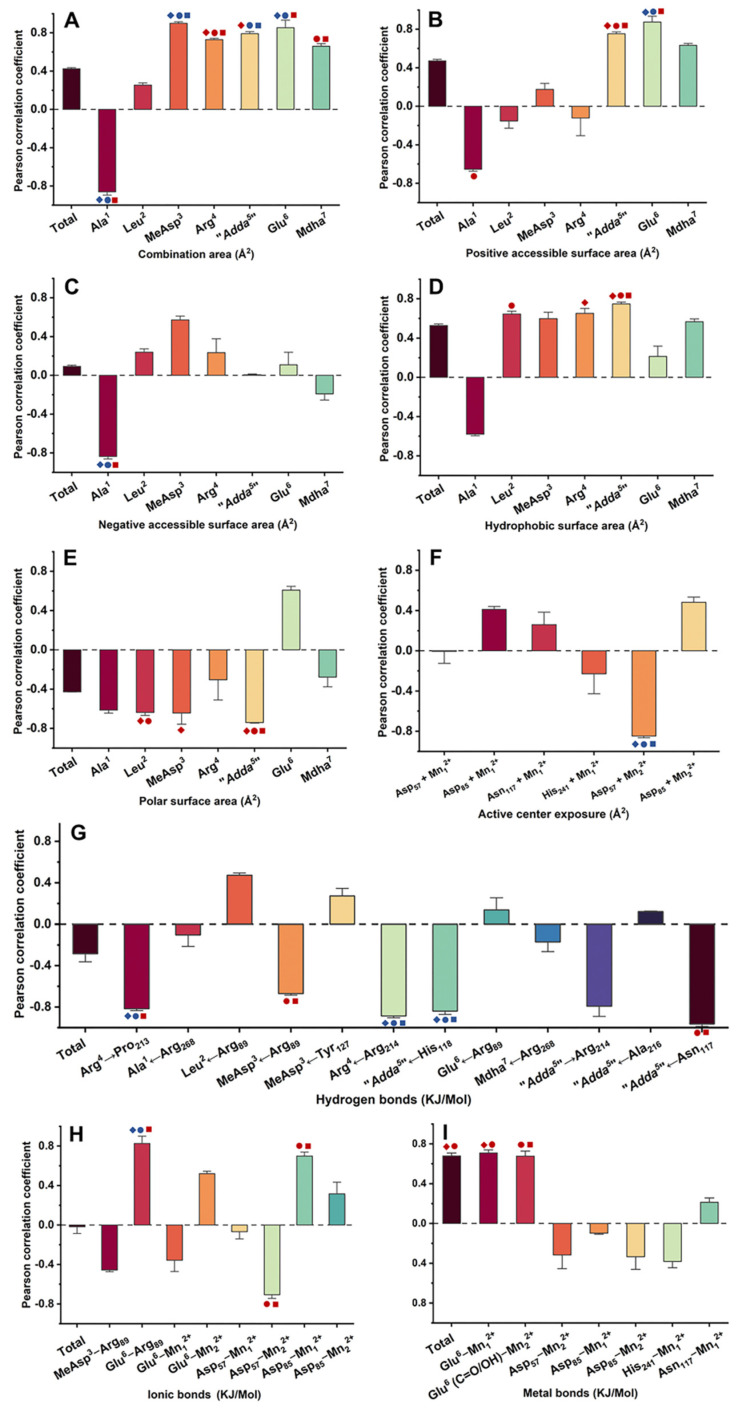
Pearson correlation coefficient between inhibition data and (**A**) combination area, (**B**) positive accessible surface area, (**C**) negative accessible surface area, (**D**) hydrophobic surface area, (**E**) polar surface area, (**F**) active center exposure, (**G**) hydrogen bonds, (**H**) ionic bonds, (**I**) metal bonds. Conditions: the interaction parameters that were significantly or highly significantly correlated with inhibition data (*p* < 0.05 or *p* < 0.01) are marked with symbols in different colors. The interaction parameters that had significantly or highly significantly correlations correlated with inhibition data at different toxin levels are marked with symbols in different shapes. 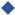, 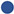, 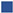 mean that the interaction parameters are extremely and significantly correlated with the inhibition data at the levels of 1, 10, and 100 nM, respectively (*p* < 0.01). 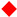, 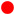, 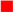 mean that the interaction parameters are significantly correlated with the inhibition data at the levels of 1, 10, and 100 nM, respectively (*p* < 0.05). A Student’s *t*-test was applied to the Pearson correlation analysis.

Pearson correlation analysis was used to evaluate the correlation between inhibition data and candidate interaction parameters. Regression analysis was not used to avoid deleting valid parameters associated with a few limited amino acid residues. According to Figure 5 and Figure S1, the interaction parameters showed diversified correlation with inhibition data: 43 interaction parameters were positively correlated with inhibition data at all or partial concentrations, while 37 interaction parameters were negatively correlated with inhibition data at all or partial concentrations; 12 interaction parameters were extremely significantly correlated with inhibition data at all or partial concentrations (*p* < 0.01), while 24 interaction parameters were significantly correlated with inhibition data at all or partial concentrations (*p* < 0.05). Among them, the interaction parameters that were significantly or extremely significantly correlated with inhibition data (*p* < 0.05 or *p* < 0.01) were crucial for the inhibition effect of MCLR and typical MCLR-DBPs on PP2A.

**Figure 6:**
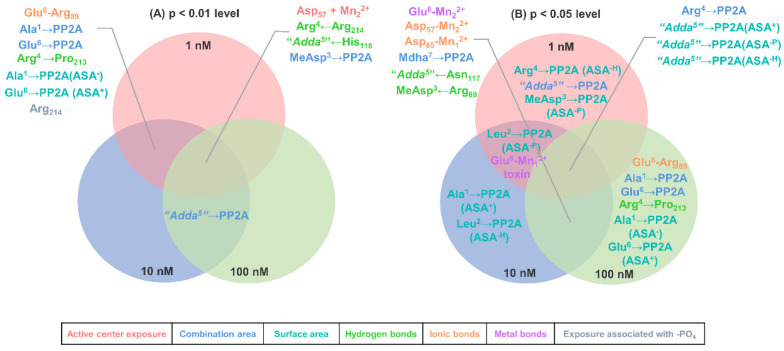
Venn diagrams of the significant interaction parameters at the *p* < 0.01 level (**A**) and *p* < 0.05 level (**B**). Conditions: ASA^+^ stands for positive accessible surface area, ASA^−^ stands for negative accessible surface area, ASA^-H^ stands for hydrophobic surface area, and ASA^-P^ stands for polar surface area.

In view of this, Venn diagrams were further used to screen the key interaction parameters (Figure 6). At the level of *p* < 0.01, the catalytic center exposure area for Asp_57_ + Mn_2_^2+^, hydrogen bonds for Arg^4^←Arg_214_, “Adda^5^”←His_118_, and the combination area for MeAsp^3^→PP2A were highly and significantly correlated with toxin toxicity at the three test concentrations. Hydrogen bond for Arg^4^→Pro_213_, ionic bond for Glu^6^-Arg_89_, the combination areas for Ala^1^→PP2A, Glu^6^→PP2A, the negative accessible surface area for Ala^1^→PP2A, the positive accessible surface area for Glu^6^→PP2A, and the amino acid associated with the binding of the phosphate group for Arg_214_ were highly and significantly correlated with toxin toxicity at 1 nM and 10 nM. The combination area for “Adda^5^”→PP2A was highly and significantly correlated with toxin toxicity at 10 nM and 100 nM. At the level of *p* < 0.05, the combination area for Arg^4^→PP2A, the positive accessible surface area for “Adda^5^”→PP2A, the hydrophobic surface area for “Adda^5^”→PP2A, and the polar surface area for “Adda^5^”→PP2A were in highly significant correlation with toxin toxicity at the three test concentrations. The metal bonds for Mn^2+^ ions to toxins, the metal bond for Glu^6^-Mn_1_^2+^, and the polar surface area for Leu^2^→PP2A were significantly correlated with toxin toxicity at 1 nM and 10 nM. Hydrogen bonds for MeAsp^3^←Arg_89_, “Adda^5^”←Asn_117_, ionic bonds for Arg_85_-Mn_1_^2+^, Arg_57_-Mn_2_^2+^, metal bond for Glu^6^-Mn_2_^2+^, and the combination area for Mdha^7^→PP2A were significantly correlated with toxin toxicity at 10 nM and 100 nM. The combination area for “Adda^5^”→PP2A, the polar surface area for MeAsp^3^→PP2A, and the hydrophobic surface area for Arg^4^→PP2A were significantly correlated with toxin toxicity at 1 nM. The positive accessible surface area for Ala^1^→PP2A and the hydrophobic surface area for Leu^2^→PP2A were significantly correlated with toxin toxicity at 10 nM. Hydrogen bond for Arg^4^→Pro_213_, ionic bond for Glu^6^-Arg_89_, the combination areas for Ala^1^→PP2A, Glu^6^→PP2A, the negative accessible surface area for Ala^1^→PP2A, and the positive accessible surface area for Glu^6^→PP2A were significantly correlated with toxin toxicity at 100 nM. Obviously, the above interaction parameters (especially those highly related to toxin toxicity at two and three test concentrations) were important for the inhibition effect of MCLR and typical MCLR-DBPs on PP2A.

**Figure 7:**
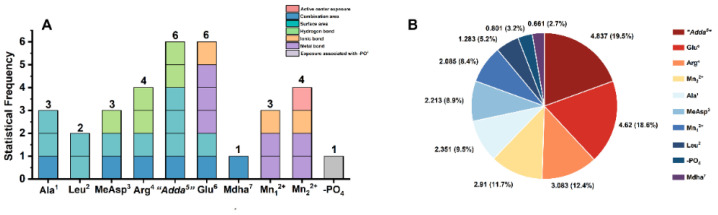
Histogram for the statistical frequency (**A**) related to the key interaction sites and pie chart for the total |$\bar{R}$| values (**B**) related to the key interaction sites. Conditions: $\bar{R}$ is the average of Pearson correlations at three toxin concentrations.

The statistical analysis was further adopted to classify the key interactions based on the structural units of MCLR/MCLR-DBPs and catalytic center (Figure 7). Among this, the metal bonds for Mn^2+^ ions to toxins should be related to Glu^6^, Mn_1_^2+^ ion, and Mn_2_^2+^ ion. Statistical frequency analysis (Figure 7A) determined that six key interaction parameters were related to “Adda^5^”/Glu^6^, four key interaction parameters were related to Arg^4^/Mn_2_^2+^, three key interaction parameters were related to Ala^1^/MeAsp^3^/Mn_1_^2+^, two key interaction parameters were related to Leu^2^, while one key interaction parameter was related to the phosphate group/Mdha^7^. Combined with statistical analysis for the total |$\bar{R}$| values related to the above sites, “Adda^5^”, Glu^6^, Arg^4^, Mn_2_^2+^ ion, Ala^1^, MeAsp^3^, Mn_1_^2+^ ion, Leu^2^, the phosphate group, and Mdha^7^ participated in the combination of MCLR/MCLR-DBPs to PP2A and their contributions exhibited a downward trend. “Adda^5^”/Glu^6^ had a prominent influence on the combination of MCLR/MCLR-DBPs to PP2A, Arg^4^/Mn_2_^2+^/Ala^1^/MeAsp^3^/Mn_1_^2+^ had a considerable influence on the combination of MCLR/MCLR-DBPs to PP2A, while Leu^2^/the phosphate group/Mdha^7^ had certain influence on the combination of MCLR/MCLR-DBPs to PP2A (Figure 7B).

### 2.5. Molecular Mechanism Analysis for the Potential Inhibition Effect of Typical MCLR-DBPs Target to PP2A

**Figure 8:**
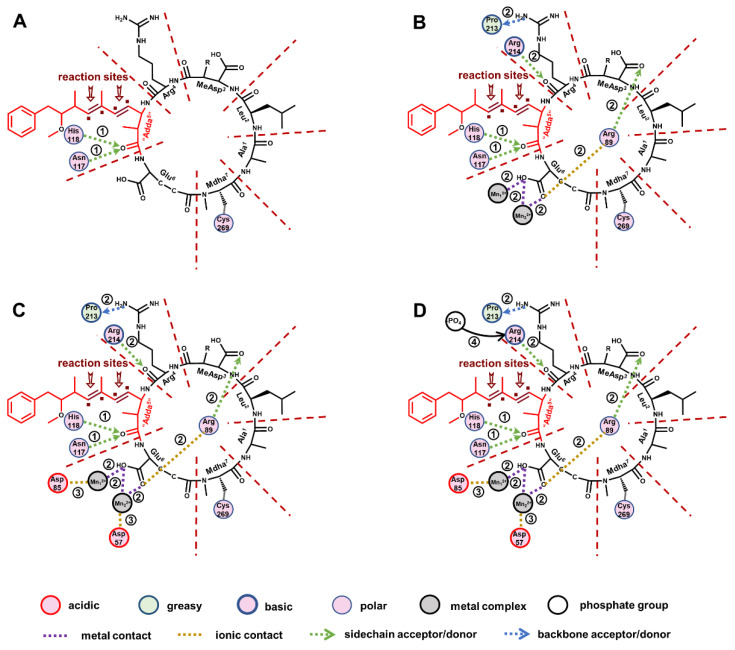
The 2D ligand–receptor interaction diagram for the combination of the toxins to PP2A. The direct influence of damaged “Adda^5^” on the interactions between toxins and PP2A (**A**). The indirect influence of damaged “Adda^5^” on the interactions between toxins and PP2A (**B**). The influence of the changed interactions on the interactions involving Mn^2+^ ions (**C**). The influence of the changed interaction on the exposure of amino acids bound to phosphate group (**D**).

The 2D ligand–receptor interaction diagram illustrated the key interactions, including hydrogen bonds “Adda^5^”←Asn_117_, “Adda^5^”←His_118_, Arg^4^←Arg_214_, Arg^4^→Pro_213_, MeAsp^3^←Arg_89_; ionic bonds Glu^6^-Arg_89_, Asp_85_-Mn_1_^2+^, Asp_57_-Mn_2_^2+^; and metal bonds Glu^6^-Mn_1_^2+^, Glu^6^-Mn_2_^2+^ (Figure 8). The identified MCLR-DBPs retained all or part of the key sites (key interactions), and thus exhibited potential inhibition effects on PP2A. With the progress of the chlorination, the structure of Adda^5^ was gradually destroyed and the inhibition effect of typical MCLR-DBPs on PP2A basically decreased as well. Obviously, structural differences changed the above key interactions and thus changed the inhibition effects of typical MCLR-DBPs on PP2A.

More specifically, the introduced polar groups ·Cl and ·OH firstly weakened the hydrophobic and electropositive interactions between “Adda^5^” and PP2A (ASA^-H^ and ASA^+^ related to “Adda^5^” were positively correlated with toxicity), resulting in the attenuated combination of “Adda^5^” to PP2A. The progressive damage of “Adda^5^” directly weakened hydrogen bonds “Adda^5^”←Asn_117_ and “Adda^5^”←His_118_ to larger degrees. The progressive damage of “Adda^5^” also intervened in the combination of other structural units of MCLR-DBPs to PP2A by weakening hydrogen bonds MeAsp^3^←Arg_89_, Arg^4^←Arg_214_, Arg^4^→Pro_213_, and by strengthening ionic bond Glu^6^-Arg_89_. Correspondingly, the combination areas of MeAsp^3^ and Arg^4^ to PP2A were decreased. However, the combination areas of Glu^6^ to PP2A did not show an increase trend. The abnormal phenomenon should be attributed to the competitive effect of Mn^2+^ ions in the catalytic center. The two Mn^2+^ ions could form new metal bonds (Glu^6^-Mn_1_^2+^, Glu^6^(C=O)-Mn_2_^2+^, Glu^6^(OH)-Mn_2_^2+^) with the side chain of Glu^6^. The above modified interactions further affected the interactions between the conserved domain of PP2A and Mn^2+^ ions (including the enhanced ionic bond Asp_85_-Mn_1_^2+^ and the weakened ionic bond Asp_57_-Mn_2_^2+^) and promoted the exposure of Mn^2+^ ions. At the same time, weakening of the hydrogen bond Arg^4^←Arg_214_ promoted the exposure areas of Arg_214_, and thus the linkage of the phosphate group to Arg_214_ was facilitated. Both the exposure of Mn^2+^ ions and the combination of Arg_214_ to the phosphate group increased, resulting in the restored catalytic activity of PP2A. 

## 3. Conclusions

This research investigated the molecular mechanism for the potential inhibition effect of typical MCLR-DBPs target to PP2A. Subject to disinfection, MCLR was oxidized into five types of typical MCLR-DBP with different molecular weights. The isomers for typical MCLR-DBPs with identical molecular weight were separated and identified as P1–P8 (mainly the ·OH/·Cl addition products related to the conjugated diene in Adda^5^). PP2A inhibition assay showed that the potential inhibition effects of toxins were in the sequence of MCLR > P1 > P4 > P3 ≈ P2 > P7 ≈ P6 ≈ P5 > P8. To elucidate the molecular mechanism underlying the inhibition effect, the interaction models for typical MCLR-DBPs-PP2A were preliminary constructed by a modeling-based-on-ligand-similarity strategy according to the crystal structure of the MCLR-PP2A complex. With the assistance of molecular docking simulation, the candidate interaction parameters between typical MCLR-DBPs and PP2A were obtained. Taking the inhibition data and candidate interaction parameters into consideration, Pearson correlation analysis filtered the key interaction parameters. The progressive damage of “Adda^5^” directly resulted in the weakening of hydrogen bonds “Adda^5^”←Asn_117_ and “Adda^5^”←His_118_, and indirectly resulted in the weakening of hydrogen bonds MeAsp^3^←Arg_89_, Arg^4^←Arg_214_, Arg^4^→Pro_213_, and the strengthening of ionic bond Glu^6^-Arg_89_. Changes in the above key interactions further affected the interactions associated with Mn^2+^ ions (in the catalytic center) by strengthening ionic bond Asp_85_-Mn_1_^2+^, metal bonds Glu^6^-Mn_1_^2+^, Glu^6^-Mn_2_^2+^, and weakening ionic bond Asp_57_-Mn_2_^2+^. The typical MCLR-DBPs retained the above key interactions, and thus exhibited potential inhibition effects on PP2A. Changes in the interactions associated with Mn^2+^ ions increased the exposure areas of Mn^2+^ ions. Meanwhile, the weakened hydrogen bond Arg^4^←Arg_214_ facilitated the linkage of the phosphate group to Arg_214_ (with increased exposure). In this way, the catalytic activity of PP2A was restored.

## 4. Materials and Methods

### 4.1. Materials

Microcystin-LR was purchased from Sigma (Saint-Quentin Fallavier, France). PP2A was obtained from New England Biolabs Inc. Na_2_S_2_O_3_, MgCl_2_, MnCl_2_, HCl, Ca(ClO)_2_, high-purity CO_2_, p-Nitrophenyldisodium orthophorphate (p-NPP), tris(hydroxymethyl)aminomethane (Tris), bovine serum albumin (BSA), dithiothreitol (DTT), neoprene rubber, sodium nitrobenzene disodium, and ascorbic acid were purchased from Sinopharm (Shanghai, China). HCOOH, CH_3_OH, CF_3_COOH, and HPLC acetonitrile were purchased from Merck (Darmstadt, Germany).

### 4.2. Chlorination Treatment of MCLR

Chlorination treatment of MCLR was performed with HClO serving as the disinfectant. HClO was prepared based on the precipitation reaction of Ca(ClO)_2_ and CO_2_ [24]. Amounts of 250 mL MCLR (100 µg/L) and 250 mL HClO (about 4 mg/L) were mixed in a 1000 mL brown reagent bottle and reacted in darkness at room temperature. At the fixed reaction time, 50 mL disinfection sample was fetched out and mixed with 5 mL ascorbic acid (about 20 mg/L).

### 4.3. Purification and Preparation of Typical MCLR-DBPs

#### 4.3.1. MS Analysis of the Disinfection Samples

The chlorination sample was mixed with the same volume of methanol (containing 0.1% formic acid) and was injected into a maXis UHR-TOF mass spectrometer for the preliminary identification of typical MCLR-DBPs. Typical MS parameters were set as follows: positive ion spray ionization pattern, source voltage 4 kV, cone voltage 0.5 kV, desolvation gas (N_2_) 0.4 bar, dry gas (N_2_) heater 180 °C, dry gas (N_2_) flow rate 4 L/min, full scan *m*/*z* 750−1200.

#### 4.3.2. Purification of Typical MCLR-DBPs

According to the traditional concentration and enrichment methods for MCs, typical MCLR-DBPs were purified [25,26]. Disinfection samples (50 mL) were applied to the pre-rinsed SepPakC_18_ SPE cartridges (1000 mg, Waters) with 10 mL methanol and 10 mL high purity water. Impurities and typical MCLR-DBPs were eluted with 5 mL 10% methanol and 5 mL 80% methanol, respectively. The crude extracts of typical MCLR-DBPs were evaporated to dryness in N_2_ flow and resuspended in 200 µL 20% acetonitrile. Then the crude extracts were separated using a Dionex Ultimate-3000 HPLC system equipped with a C_18_ reversed-phase preparative column (25.4 mm × 450 mm, 5 µm, 120 Å) [21]. Water (containing 0.1% formic acid) and acetonitrile (containing 0.1% formic acid) were used as mobile phase A and mobile phase B, respectively. The elution conditions were: 20% mobile phase B for 3 min; 20%→80% mobile phase B over 25 min; 80%→20% mobile phase B within 0.1 min; 20% mobile phase B for 3 min. The column temperature and the flow rate were set at 35 °C and 5 mL/min, respectively.

#### 4.3.3. Preparation of Typical MCLR-DBPs

At the same time, partial chromatographic effluent was guided into the maXis UHR-TOF mass spectrometer through a four-way valve with the assistance of an autosampler. MS parameters were set as those of Section 4.3.1 except “full scan” mode was changed to “selective ion scan” mode. The specific retention times for typical MCLR-DBPs (especially for the isomers) could be obtained. Chromatography-separated typical MCLR-DBPs were collected at their specific retention times and separately stored in brown reagent bottles [21]. Multi-collect pure samples for typical MCLR-DBPs were dried with N_2_ and dissolved in 200 µL methanol. Based on MS/MS analysis, the prepared MCLR-DBPs were identified by comparing their secondary structures with that of MCLR (MS/MS parameters were set as those of Section 4.3.1 except N_2_ collision gas energies were adjusted from 40 to 100 eV, and full scan range was adjusted as *m*/*z* 100−1200).

### 4.4. PP2A Inhibition Assay for MCLR and Typical MCLR-DBPs

The inhibition effect of typical MCLR-DBPs on PP2A was evaluated with a typical protein phosphatase inhibition assay [27]. First, PP2A was diluted to 5 U/mL with buffer solution (50 mM Tris-HCl, 1.0 mM MnCl_2_, 2.0 mM dithiothreitol, pH 7.4) and 1.0 g/L BSA. Afterwards, 10 µL PP2A and 100 µL samples were mixed into a 96-well polystyrene microplate plate. After shaking slightly, the microplate was kept at 25.0 °C for 15 min and 90 µL p-NPP was added to the microtiter plate. After 60 min, absorbance ODS_405_ was measured with a microplate reader. The PP2A relative activity percentage formula is as follows: I_PP2A_ (%) = (A_toxins_ − A_blank_)/(A_control_ − A_blank_) × 100%. In the control group, toxins were replaced by distilled water, and in the blank group MCLR/MCLR-DBPs and PP2A were replaced by distilled water.

### 4.5. Molecular Simulation for the Interactions between MCLR/MCLR-DBPs and PP2A

Molecular simulation was performed with Molecular Operating Environment software (MOE, version 20.09). The experimental steps were as follows: The model for MCLR-PP2A was obtained from Protein Data Bank (PDB code 2IE3). When the model for the MCLR-PP2A complex was introduced into MOE, MCLR and PP2A were preprocessed by “building missing loops” and adjusting the charges of the whole system [28]. The models for MCLR-DBPs-PP2A were preliminarily constructed by modeling based on ligand similarity: the original ligand MCLR in the optimized model of MCLR-PP2A was replaced by different MCLR-DBPs [22,28]. The models for MCLR-DBPs-PP2A complexes were energy-minimized to determine the optimal interaction geometry and associated energy between MCLR-DBPs and PP2A and to check the rationality of crystal structures. Subsequently, the optimized models for MCLR-DBPs-PP2A complexes were re-docked though “template dock” mode and then the interactions between MCLR/MCLR-DBPs and PP2A were simulated. The “template dock” mode developed by MOE software was suitable for binding sites whose locations are known but information about specific ligand interactions is lacking, which ensured the comparability of MCLR-DBPs with their original toxin [22]. Docking parameters were set as follows: amber 10 EHT, solvation r-field, temperature 25.0 °C, pH 7.4, salinity 0.05 M. The candidate interaction parameters (combination areas, related surface areas, hydrogen bonds, metal bonds, ionic bonds, exposure areas of the catalytic center) related to the combination of toxins to PP2A could be obtained.

### 4.6. Statistical Analysis

Pearson correlation analysis was used to analyze the correlations between inhibition data and candidate interaction parameters with IBM SPSS Statistics software (version 26.0, Chicago, IL, USA). Hypothesis testing of Pearson correlation coefficient was performed with Student’s *t*-test. Significance levels are reported to be highly significant (*p* < 0.01), significant (*p* < 0.05), or not significant (*p* > 0.05). One-way analysis of variance (ANOVA) followed by least significant difference (LSD) post-hoc tests was used to check significant differences among groups and *p* < 0.05 values were considered statistically significant.

## Data Availability

Not applicable.

## Supplementary Materials

The following supporting information can be downloaded at: https://www.mdpi.com/article/10.3390/toxins14120878/s1, Table S1: MS/MS identification of MCLR and MCLR-DBPs; Table S2: Preparation and purification information for typical MCLR-DBPs; Table S3: The candidate interaction parameters between MCLR/MCLR-DBPs and PP2A; Figure S1: Pearson correlation coefficients between inhibition data and exposure areas associated with phosphate group.
