# Supplementary material for: Mechanism for the Potential Inhibition Effect of Microcystin-LR Disinfectant By-Products on Protein Phosphatase 2A
Source: Toxins (Basel). 2022 Dec 16;14(12):878. doi: 10.3390/toxins14120878 (PMC9780900; doi:10.3390/toxins14120878)
Supplement: Supplementary file 1 [file toxins-14-00878-s001.zip › toxins-1934819-supplementary.pdf]

Table S1. MS/MS identification of MCLR and MCLR-DBPs

Basic fragment ions	MCLR	$C_{34}H_{54}N_{10}O_{12}$ (P8)	$C_{49}H_{76}N_{10}O_{14}Cl_2$ (P5)	$C_{49}H_{77}N_{10}O_{15}Cl$		$C_{49}H_{75}N_{10}O_{13}Cl$		$C_{49}H_{76}N_{10}O_{14}$	
				P6	P7	P1	P2	P3	P4
$[M+H]^+$	995.5559	795.3995 ↓ [a]	1099.4991 ↑	1081.5331 ↑	1081.5331 ↑	1047.5276 ↑	1047.5276 ↑	1029.5616 ↑	1029.5616 ↑
$[Glu\text{-}Mdha+H]^+$	213.0834	213.0835 √ [b]	213.0834 √	213.0831 √	213.0833 √	213.0833 √	213.0832 √	213.0836 √	213.0838 √
$[MeAsp\text{-}Arg+H]^+$	286.1479	286.1477 √	286.1481 √	286.1476 √	286.1478 √	286.1481 √	286.1482 √	286.1481 √	286.1482 √
$[Mdha\text{-}Ala\text{-}Leu\text{-}MeAsp\text{-}Arg+H]^+$	553.3072	553.3070 √	553.3071 √	553.3070 √	553.3070√	553.3073 √	553.3072 √	553.3067 √	553.3069 √
$[MeAsp\text{-}Arg\text{-}Adda+H]^+$ or $[Arg\text{-}Adda\text{-}Glu+H]^+$	599.3546	399.1993 ↓	703.2987 ↑	685.3326 ↑	685.3327 ↑	651.3268 ↑	651.3269 ↑	633.3606 ↑	633.3608 ↑
$[Arg\text{-}Adda\text{-}Glu\text{-}Mdha+H]^+$	682.3959	482.2856 ↓	786.3361 ↑	768.3698 ↑	768.3699 ↑	734.3639 ↑	734.3639 ↑	716.3978 ↑	716.3977 ↑
$[Mdha\text{-}Ala\text{-}Leu\text{-}MeAsp\text{-}Arg–Adda+H]^+$ or $[Arg\text{-}Adda\text{-}Glu\text{-}Mdha\text{-}Ala\text{-}Leu+H]^+$	866.5150	666.3568 ↓	970.4566 ↑	952.4906 ↑	952.4906 ↑	918.4858 ↑	918.4858 ↑	900.5189 ↑	900.5190 ↑
Target residues	---	Adda							

[a]: ↑ and ↓ mean mass changes were related to these fragment ions; [b]: √ means ions with the stable m/z were detected by mass spectrograph.

Table S2. Preparation and purification information for typical MCLR-DBPs

MCLR-DBPs	Disinfection times	LC collection times	Concentration [a]	Purity [b]
P1	20, 30, 40, 50, 60 mins	21.67± 0.25 min	32.4 μmol/L	98.6%
P2	20, 30, 40, 50, 60, 70 mins	22.29± 0.25 min	41.6 μmol/L	99.2%
P3	30, 40, 50, 60, 70, 80, 90 mins	17.44± 0.15 min	103.2 μmol/L	99.6%
P4	30, 40, 50, 60, 70, 80, 90 mins	17.85± 0.15 min	128.3 μmol/L	99.1%
P5	20, 30, 40, 50 mins	27.44 ± 0.25min	22.5 μmol/L	99.4%
P6	20, 30, 40, 50, 60 mins	19.48± 0.25 min	49.2 μmol/L	98.7%
P7	20, 30, 40, 50, 60 mins	20.09± 0.25 min	20.8 μmol/L	99.3%
P8	20, 30, 40, 50 mins	16.82± 0.25 min	52.8 μmol/L	99.2%

[a:] The concentrations of typical MCLR-DBPs were directly calculated according to their MS signals compared with that of MCLR. MCLR and MCLR-DBPs were assumed had approximate protonated efficiencies.

[b:] The purity of typical MCLR-DBPs was directly calculated according to their MS intensities and defined as $I_{Pi}/(I_{MCLR}+\sum I_{Pi}) \times 100\%$.

Table S3. The candidate interaction parameters between MCLR/MCLR-DBPs and PP2A

Molecular simulation parameters		MCLR	P1	P2	P3	P4	P5	P6	P7	P8
Combination energy (KJ/Mol)		-5388.70	-5343.66	-5391.47	-5385.90	-5350.71	-5344.69	-5336.77	-5347.76	-5358.97
logP (o/w)		-77.5350	-78.0770	-77.3720	-78.6200	-79.3250	-77.9140	-79.1620	-79.1620	-81.4850
logS		-249.6839	-249.1139	-249.7440	-248.7762	-248.1461	-249.8009	-248.8331	-248.8331	-246.0052
Combination area (Å^2)	Total	698.0694	691.0163	690.4417	692.5611	695.8382	683.0987	688.6780	684.5360	487.6997
	Ala^1→PP2A	51.3982	53.3917	59.0195	59.7478	60.5655	61.3226	61.8029	62.0973	61.9705
	Leu^2→PP2A	141.2107	141.7950	140.0439	140.464	140.4281	139.4582	140.7596	140.6998	142.0348
	MeAsp^3→PP2A	68.6157	68.1460	66.8960	66.9288	67.3469	64.7011	64.9361	64.7248	68.2037
	Arg^4→PP2A	111.0707	110.4977	110.3533	109.4538	105.0008	103.7783	106.8322	106.0855	104.7621
	Adda^5→PP2A	374.1398	366.9286	362.2490	362.9485	364.4504	353.5948	353.0048	354.9035	162.4581
	Glu^6→PP2A	170.7896	166.3016	164.2712	165.9308	165.3219	163.5029	164.2210	163.7905	164.3255
	Mdha^7→PP2A	122.1555	122.1173	122.2674	122.3941	122.4282	120.9693	118.3658	118.6729	118.0450
Positive accessible surface area (Å^2)	Total	439.1316	435.6923	430.8363	437.7828	432.8109	420.4103	424.9225	425.8085	304.6616
	Ala^1→PP2A	26.4509	27.9869	28.4739	28.8025	29.2864	29.2506	30.4338	30.7539	30.2222
	Leu^2→PP2A	90.0988	90.2805	92.2072	91.8388	91.7188	90.2699	90.9062	90.8842	90.3118
	MeAsp^3→PP2A	39.2427	38.2746	37.7506	38.1432	35.5680	35.9824	36.0599	35.2263	40.6580
	Arg^4→PP2A	76.1616	61.3198	72.6042	73.0512	67.0054	68.4859	70.8275	70.8242	72.6749
	Adda^5→PP2A	252.6611	238.5448	215.5528	218.9282	232.8600	203.1143	202.1682	208.7413	126.6315
	Glu^6→PP2A	95.3726	90.8768	89.9431	90.1072	89.4909	87.3470	88.1905	88.1352	86.0387
	Mdha^7→PP2A	84.5584	84.6814	83.3083	83.3586	83.0723	82.5923	80.0678	80.4473	81.3753
Negative accessible surface area (Å^2)	Total	251.3508	254.3241	261.6056	252.7785	251.5278	275.6880	272.7560	270.7278	183.0384
	Ala^1→PP2A	24.9473	25.4045	30.5460	30.9452	31.2296	32.0720	31.3699	31.3429	31.7483

	Leu^2→PP2A	51.1115	51.5144	47.8372	48.6252	48.7095	49.1885	49.8542	49.8154	51.7230
	MeAsp^3→PP2A	29.3731	30.8713	28.6459	28.5860	28.7789	28.7189	28.8764	28.9982	25.5459
	Arg^4→PP2A	34.9089	37.1777	37.7491	36.4026	36.9956	35.2924	36.0054	35.2610	34.0873
	Adda^5→PP2A	122.4788	123.3841	137.6964	127.0203	124.5904	149.7369	154.9806	148.6617	55.8266
	Glu^6→PP2A	78.4167	73.9248	74.3285	75.8238	75.8310	75.1561	76.0311	75.6552	78.2868
	Mdha^7→PP2A	37.5967	37.4362	38.9597	39.0353	39.3560	38.3769	38.2987	38.2253	37.6697
Hydrophobic surface area (Å^2)	Total	415.0878	403.5900	391.1444	395.1850	401.2825	384.3636	385.7173	382.6232	225.5066
	Ala^1→PP2A	9.1599	9.0779	11.9868	12.7465	12.7449	14.0165	14.1496	14.0117	13.7089
	Leu^2→PP2A	60.8376	60.3547	58.0986	58.0835	57.5584	58.3567	57.9787	58.7842	53.2171
	MeAsp^3→PP2A	8.7165	6.9260	5.1297	4.8409	3.3302	3.0175	3.0157	2.8326	6.5986
	Arg^4→PP2A	43.0193	37.7420	40.9852	40.3010	41.9806	38.4102	37.7660	37.9445	37.0601
	Adda^5→PP2A	272.5860	248.9771	220.9143	221.2025	243.4168	208.4423	201.1964	211.5261	98.4271
	Glu^6→PP2A	49.1993	45.2458	45.5970	47.2925	47.8322	46.7353	46.4343	46.8372	48.8208
	Mdha^7→PP2A	88.4628	87.4585	85.8546	86.5487	86.1776	85.7024	83.9827	84.2291	83.7327
Polar surface area (Å^2)	Total	275.3944	286.4260	301.2973	295.3763	282.0559	311.7350	311.9608	313.9125	262.1935
	Ala^1→PP2A	42.2155	44.3131	47.0332	47.0011	47.7709	47.3063	47.6537	48.0851	48.2618
	Leu^2→PP2A	80.3728	81.4399	81.9455	82.3804	81.8697	81.1017	82.7813	81.9153	84.8178
	MeAsp^3→PP2A	59.8991	62.2195	61.7666	61.8882	61.0166	61.6837	61.9203	61.8918	61.6053
	Arg^4→PP2A	71.0511	61.7554	68.3681	67.1528	62.0200	67.3686	68.0666	68.1404	70.7022
	Adda^5→PP2A	112.5535	120.0512	141.3350	134.7460	126.0332	156.6530	154.7085	153.8768	114.0312
	Glu^6→PP2A	121.7466	121.0555	118.6547	118.6384	117.4894	116.7680	117.7867	116.9534	115.5049
	Mdha^7→PP2A	33.6925	34.6584	36.4132	35.8450	36.2505	35.2671	34.3835	34.4437	35.3122
	Total	-43.1	-40.1	-43.8	-42.9	-41.0	-40.8	-40.0	-41.8	-40.2

Hydrogen bonds (KJ/Mol)	Arg^{4}→Pro$_{213}$	-7.0	-6.1	-6.3	-6.4	-6.4	-5.4	-5.6	-5.3	-5.1
	Ala^{1}←Arg$_{268}$	-2.6	-2.3	-1.8	-1.9	-1.9	-2.4	-2.3	-2.4	-2.4
	Leu^{2} ←Arg$_{89}$	-2.2	-2.1	-2.3	-2.2	-2.4	-2.5	-2.4	-2.5	-4.4
	MeAsp^{3}←Arg$_{89}$	-6.6	-6.4	-6.3	-6.3	-6.6	-6.1	-6.2	-6.3	-4.3
	MeAsp^{3}←Tyr$_{127}$	-1.6	-1.7	-1.7	-1.9	-2.4	-2.2	-2.1	-2.2	-1.7
	Arg^{4}←Arg$_{214}$	-6.6	-6.4	-6.2	-6.2	-6.4	-6.1	-6.1	-6.1	-5.7
	Adda^{5}←His$_{118}$	-1.6	-1.4	-1.4	-1.4	-1.5	-1.4	-1.3	-1.3	-1.2
	Glu^{6}←Arg$_{89}$	-8.2	-7.1	-7.1	-7.3	-7.5	-7.7	-7.7	-7.6	-8.8
	Mdha^{7}←Arg$_{268}$	-6.7	-6.6	-6.6	-6.4	-5.9	-6.4	-6.3	-6.0	-6.6
	Adda^{5}→Arg$_{214}$	---[a]	---	-2.6	-2.0	---	---	---	-1.1	---
	Adda^{5}← Ala$_{216}$	---	---	-0.7	---	---	---	---	---	---
	Adda^{5}←Asn$_{117}$	---	---	-0.8	-0.9	---	-0.6	---	-1.0	---
Ionic bonds (KJ/Mol)	Total	-109.8	-111.0	-111.5	-112.3	-112.0	-112.2	-110.7	-112.1	-107.6
	MeAsp^{3}-Arg$_{89}$	-7.7	-7.8	-7.6	-7.7	-7.5	-6.9	-7.5	-7.4	-3.0
	Glu^{6}-Arg$_{89}$	-6.9	-7.3	-7.3	-7.6	-7.3	-7.5	-7.5	-7.4	-7.5
	Glu^{6}-Mn_1^{2+}	-12.1	-12.5	-12.2	-12.2	-12.4	-12.3	-12.2	-12.3	-11.7
	Glu^{6}-Mn_2^{2+}	-22.8	-22.9	-23.1	-23.5	-23.6	-23.4	-22.9	-23.7	-24.6
	Asp$_{57}$-Mn_1^{2+}	-12.2	-12.3	-12.5	-12.5	-12.5	-12.4	-12.0	-12.5	-12.1
	Asp$_{57}$-Mn_2^{2+}	-14.9	-14.9	-14.9	-14.9	-14.9	-14.8	-14.8	-14.8	-14.7
	Asp$_{85}$-Mn_1^{2+}	-21.0	-20.9	-21.4	-21.4	-21.4	-22.5	-21.5	-21.6	-21.7
	Asp$_{85}$-Mn_2^{2+}	-12.2	-12.4	-12.5	-12.5	-12.4	-12.4	-12.3	-12.4	-12.3
Metal bonds (KJ/Mol)	Total	-42.2	-42.5	-44.0	-43.2	-43.2	-43.2	-43.6	-42.9	-43.2
	Glu^{6}-Mn_1^{2+}	-4.2	-4.6	-4.8	-4.8	-5.0	-5.0	-5.2	-4.8	-6.0

	Glu^{6}-Mn_2^{2+}	-9.2	-8.8	-9.5	-9.4	-9.3	-9.3	-9.5	-9.4	-9.9
	Asp_{57}-Mn_1^{2+}	-5.3	-5.3	-5.3	-5.3	-5.4	-5.3	-5.3	-5.3	-5.3
	Asp_{57}-Mn_2^{2+}	-5.7	-5.7	-5.7	-5.7	-5.7	-5.7	-5.7	-5.7	-5.7
	Asp_{85}-Mn_1^{2+}	-6.8	-6.5	-7.1	-7.1	-6.9	-7.2	-7.2	-7.2	-5.0
	Asp_{85}-Mn_2^{2+}	-4.7	-4.8	-4.8	-4.8	-4.9	-4.8	-4.7	-4.7	-4.6
	His_{241}-Mn_1^{2+}	-2.0	-2.2	-2.2	-2.2	-2.1	-1.5	-1.6	-1.4	-2.2
	Asn_{117}-Mn_1^{2+}	-4.3	-4.6	-4.6	-3.9	-3.9	-4.4	-4.4	-4.4	-4.5
Exposure areas associated with -PO_4 (Å^2)	Arg_{89}+His_{118}+Arg_{214}	1001.8087	1002.3587	954.2913	924.2706	922.9355	1000.8712	1001.4561	1002.5461	998.4578
	Arg_{89}	374.1155	376.3034	377.4064	375.9305	376.6839	372.9275	375.6780	374.7650	370.4567
	His_{118}	319.8264	313.1279	315.9004	319.5454	320.8376	318.6528	317.5461	315.9704	313.5746
	Arg_{214}	322.0467	374.2768	375.9326	378.0033	381.4669	382.916	388.9326	387.0086	395.2576
Active center exposure (Å^2)	Asp_{57} + Mn_1^{2+}	314.8653	317.6787	316.2990	315.0376	319.1743	316.2121	315.4156	315.6590	319.0143
	Asp_{85} + Mn_1^{2+}	310.2860	311.5403	307.4261	308.8214	308.6803	306.9382	311.4167	308.9003	308.6871
	Asn_{117} + Mn_1^{2+}	322.4064	321.9360	321.7552	322.9602	326.6022	321.1173	321.5983	322.5563	323.6511
	His_{241} + Mn_1^{2+}	356.1933	352.6238	354.9702	354.2555	352.9492	357.6321	354.5722	355.0291	354.3359
	Asp_{57} + Mn_2^{2+}	311.4394	312.0476	312.2896	312.9623	312.7266	313.7292	313.0031	313.7058	313.8454
	Asp_{85} + Mn_2^{2+}	319.8060	320.0476	317.5977	317.6063	318.1689	306.9382	319.6428	318.2084	315.0497

[a:] --- no related parameter was detected

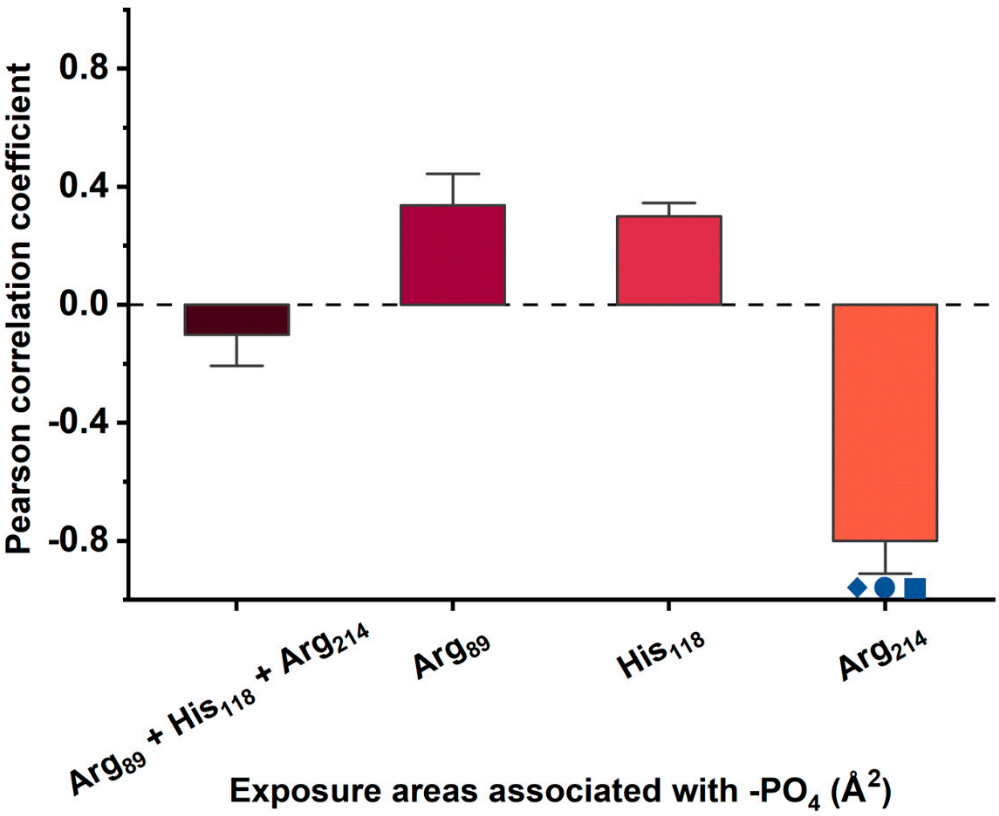

Figure S1. Pearson correlation coefficients between inhibition data and exposure areas associated with phosphate group.
